# Notes and News

**Published:** 1903-05-30

**Authors:** 


### Notes and News.

The Princess Christian presided at the meeting of the National Health Society on Saturday. The chief discussion related to infant mortality, and the Bishop of Stepney, Dr. Dudfield, and Dr. Dawson Williams spoke on the subject. It was announced that the Society had arranged for a course of lectures in reference to plumbers' work, the first lecture of the series to be given at the Mansion House.

In the proposal relating to old age pensions which has been occupying the attention of Parliament, the stipulations which would debar those who have received Poor Law relief from receiving pensions do not apply to sick relief. This would materially affect the attitude of the poor towards sickness assurance, a form of thrift that has become more universally adopted every year. If the money now paid for this is otherwise saved no harm will be done, but the instinct to save for old age is not so strong as that which leads working men to secure a living for themselves and family when they are overtaken by illness or accident. The premiums required are so small for a possible substantial payment that sickness assurance has a far greater attraction than any other.

The foundation-stone of the Winsley Sanatorium for Consumption, which is to be erected by the Gloucester, Somerset and Wilts branch of the National Association for the Prevention of Consumption, was opened on Thursday by Lady Dickson Poynder. The movement has created much interest in the counties concerned, and a large sum of money has been collected already. The architects are Messrs. Silcock and S. Reay, of Bath. The plan adopted is a long narrow block, one room deep, with a corridor immediately behind, and of two storeys in height. The walls opposite to the windows in the patients' rooms and above the level of the door heads are formed of casements and frames, opening upon centres, so that complete cross-ventilation may be obtained.

Small-pox is not yet wholly absent from the Metropolis, upwards of sixty patients being under treatment last week. Some cases are also reported from the Harrow district. A case of small-pox occurred on the *Majestic* on her present outward voyage and was removed at Queenstown.

Owing to the great interest excited by the visit of the King and Queen to St. Paul's Cathedral on June 7th, we are requested to state that tickets may be obtained, and subscription lists to the Hospital Sunday Fund seen, at the offices of the Fund, 18 Queen Victoria Street, E.C., from the hours of 12 to 4 each day.

The result of the Paris stockbrokers' walking contest, if not revealing the powers of endurance shown by the English rivals, was no mean performance. The temperature was far more trying and must have been especially so to those not previously hardened by an athletic education. M. Bouveur, the winner of the contest, walked the 25¼ miles of the route in 4¾ hours, which constituted a very respectable average of speed to maintain for nearly five hours on a hot day.

Everyone will agree with Lord Londonderry, who, when speaking at Clerkenwell, advocated the physical improvement of the young of this nation. But all will not be equally sanguine as he is that physical education will effect the remedy. It is a question whether the small white-faced, underfed, and badly-housed child (so typical of a large proportion of the population in the crowded districts where the poor congregate) would be the better, even for the most scientific physical training, so long as it is without both fresh air and proper food. The life of many of the children in the poorest districts is accompanied by an endurance of more than a due amount of physical fatigue as it is. The hours they keep are both early and late, and although regulated exercise would undoubtedly be useful as a moral education, it is doubtful if any very great physical improvement can be expected for the East End child by the means of exercise alone.

An investigation of the aerated water manufacturers in London has been made for the County Council, and the result is very disquieting. Numbers of people drink aerated waters in preference to natural water under the belief that the water used in the manufactured article is purer. The investigation shows that many of these aerated waters are subject to much possible pollution. The public have only to glance casually at the majority of soda-water syphons in use to convince them they are uncleanly. There are certain manufacturers who pride themselves on the cleanliness and purity of their beverages and methods. But these qualities are especially advertised, and high prices are charged. The public might draw an inference from this, and either submit to the larger charge, or avoid mineral waters until greater security is obtainable. The humble "sparklet" and the much improved water of the metropolis is left to them after all.

